# Interest-driven and legal supervision innovation of the capital pool model in Chinese banking financial products

**DOI:** 10.1016/j.heliyon.2023.e16181

**Published:** 2023-05-11

**Authors:** Yilin Hu

**Affiliations:** Sydney Law School, The University of Sydney, Sydney, City Rd, Sydney, 2006, Australia

**Keywords:** Bank financing, China, Capital pool model, Rigid payment, Shadow banking, “The new regulations on asset management”

## Abstract

This paper studies the formation, interest-driven rationality and potential risks of the capital pool model in China's banking financial management business, as well as the correlation, coincidence and complexity of fund pool prohibition and the rigid payment strategy. Focusing on “The New Regulations on Asset Management” issued by the Chinese government in April 2018, this paper discusses the regulatory effects and existing problems of fund pool prohibition and rigid payment regulations. From the perspective of theoretical and empirical analysis, this paper studies the impact of the relationship between financial product yield and regulatory interest rate relationship on shadow banking. The paper also studies the capital pool model that is closely related to the shadow banking, rigid payment and unstandardized debts, so as to puts forward relevant policy suggestions on improving the external regulation and optimizing the internal control mechanism of shadow banking. This paper puts forward that pursuit of financial security value should not be independent from the development of the overall interests of asset management market. The reasonable and healthy development of the asset management industry should be guided by the principle of controlling the risks at an appropriate level. The regulations in relation to capital pool and rigid payment needs more flexibility and elasticity to help reduce or eliminate the negative impact on the efficiency of resource allocation in the asset management industry. Shadow banking plays an important role in the financing of small and medium-sized enterprises, which is the result of mutual competition and game between different banks in terms of yield rate. Moderate shadow banking scale plays a positive role in the macro economy. This argument has theoretical value and practical significance to ensure that the regulatory system is resilient to the financial system in the most effective way possible.

## 1 Introduction

When enterprises cannot get money through formal bank channels, shadow banking becomes an important financing channel, which satisfying the financing function that the commercial bank credit cannot provide. It is an alternative and supplement mechanism to the formal credit mechanism. However, due to the lack of necessary regulations, shadow banking becomes an important channel of commercial banks to avoid regulations, and therefore easy to become a major hidden danger of systemic risk. Its financing vulnerability would increase the instability of the financial system. Shadow banking business can promote the increase of capital return of commercial banks, but it will cause the decline of its own stability [1]. “Shadow banking is the product of arbitrage behavior, which has the dual characteristic of angel and devil” [2]. The complex capital structure formed by shadow banking and other financial institutions easily enables the financial risks to rapidly transmit through the whole financial system [3]. Strengthening the supervision of shadow banking has been widely recognized by the international community, and it has also become a key policy issue for maintaining financial stability [4]. At present, China's supervision of shadow banking is still in the stage of exploration and improvement. To some extent, there is a lack of supervision, incomplete laws, opaque market information etc., which makes it difficult to effectively regulate shadow banking and its complex transactions and activities. Shadow banking and cross-financial business cause local risks and hidden dangers. The behavior of Chinese commercial banks in avoiding liquidity regulations has promoted the rise of shadow banking, and the pressure of bank performance and deposit competition also explain the endogenous mechanism of China's shadow banking. Therefore, how to prevent and defuse the risks of the shadow banking system and meet the needs of the real economy at the same time, and hot to effectively guide the expansion of the shadow banking scale have become two important issues to be solved urgently. “The New Regulations on Asset Management” largely reduce the possibility and severity of future crises. But the assessment on its effectiveness and its impact on loans to small and medium-sized enterprises is essentially a forward-looking work. The implementation of “The New Regulations on Asset Management” will produce joint effect on China's overall economy. It is necessary to objectively analyze effect of its the implementation and potential issues, especially the correlation with the shadow banking. This is to help regulators correctly evaluate its policy implementation effect and improve the regulatory method. This has great theoretical significance and practical value, and is also one of the difficult problems that need to be solved urgently in the development of capital pool financial products.

With the increase in residents' wealth and the demand for wealth management, China's capital management market has grown explosively. By the end of 2019, the balance of funds raised by Chinese financial institutions' existing capital management products was RMB 79.4 trillion. Among them, bank financial products account for 30.5% [5]. Commercial banks have a broad client base and sufficient funds. They have great advantages in carrying out their financial management business and promoting the development of the real economy, and they have played an important role in enhancing bank profitability, optimizing economic well-being and improving the structure of market financing. However, because of the maturity mismatch, bank financial management with the capital pool as the mainstream model leads to income mismatch and rigid payment. These have accumulated industry risks and involve difficulties in supervision and governance. As scholars and institutions have proposed, the operation of the capital pool model, maturity mismatch and rigid payments to clients with high interest rates have become characteristic of Chinese “bank financing” [6]. The “capital pool” business model and rigid payment strategy become the source of industry risk accumulation [7]. The rigid payment of the debt side has been a chronic disease in the Chinese asset management industry for many years [8]. The asset management industry still has a significant maturity and liquidity mismatch [9]. Although maturity mismatches have made banks more profitable, they also bring liquidity risk, interest rate risk and credit risk [10], which has resulted in the formation of a large Chinese shadow bank^1^ [11] with potential systemic risks. The base of these risks is difficult to measure and needs to be constrained and changed.

On April 27, 2018, the People's Bank of China, the China Banking and Insurance Regulatory Commission, the China Securities Regulatory Commission and the State Administration of Foreign Exchange jointly issued guidance on Regulating the Asset Management Business of Financial Institutions (No 0.106 [2018], For short, “The New Regulations on Asset Management”). The document offers important guidance for preventing systemic financial risks and comprehensively regulating the asset management business of various financial institutions since the reform of China's financial system. It has opened the era of unified supervision of “big asset management”, that is, developing from separate supervision to mixed supervision. It is also a regulation that responds to the problems and risks arising from asset management practice. Its core content bans the capital pool model, breaks down the rigid payment strategy, and changes the net value of the financial products. However, the new regulations have many defects, and the implementation process is complex. As a result, the actual impact and regulatory effect remain subject to market evaluation.

To ensure a smooth transition and account for the duration and scale of stock capital management products and the assets invested, “The New Regulations on Asset Management” put forward the principle of “dividing the new and the old” and set a transition period. The transition period extends “from the date of publication of the Opinions to the end of 2020". On July 31, 2020, the People's Bank of China officially announced the extension of the transition period of “The New Regulations on Asset Management” to the end of 2021. The extension of the transition period does not touch upon changes and adjustments in relevant regulatory standards [12]. The extension of the transition period gives asset management organizations sufficient time to dispose of assets that are inconsistent with the new regulations and relieves the pressure of centralized disposal.

Since the implementation of “The New Regulations on Asset Management”, the number of bank financial products issued and the scale of raising funds have shown a downward trend [13]. The growth momentum and potential systemic risks of financial products with rigid payment have been contained, and the disorderly development of shadow banking has been effectively managed [14]. This is also the most direct impact of “The New Regulations on Capital Management” on bank financing. However, the problems of “difficult financing and expensive financing” in the real economy have intensified accordingly. After the new rules strictly prohibit financial products from maturity mismatches and capital pools, investing in nonstandard assets can only issue long-term financial products, which is more difficult. The decline in the transformation efficiency of savings and investment has caused difficulties in the endogenous capitalization of existing stock currencies. Small and medium-sized private enterprises have difficulty obtaining bank financing, and they face further weakened access to off-balance-sheet financing. The decline in nonstandard investment has dragged down China's economic growth. Scholars believe that the implementation of “The New Regulations on Asset Management” has hindered the realization of the capitalization of stock currency. The internal currency circulation mechanism was destroyed [15]; the original shadow banking financing model of enterprises was unsustainable after the implementation of the new regulations, the financing difficulties of some enterprises were prominent, and the social financing system was greatly affected [16]. “The New Regulations on Asset Management” put the market in a state of anxiety and unease. Some voices even called for “giving up the new regulations”. Currently, bank financing is still not separate from the operation of the capital pool model. In 2021, there were still 13.27 trillion yuan of financial products facing net worth transformation and rectification [17]. Regulatory authorities have also carried out industry risk investigations many times, especially focusing on key rectification areas such as nonstandard capital pools and channel businesses [18]. The reasons for the slow progress in prohibiting the capital pool model do not stem from the inaction of banks or the inadequate implementation of supervision but from deep-seated economic factors. How banks undertake nonstandard assets is a difficult problem. In recent years, dealing with the large number of financial products in the capital pool model has become an important consideration for Chinese regulatory authorities. The reason is that the success of the transformation of bank financial management is of great significance in the implementation of “The New Regulations on Asset Management”.

The accumulated problems and contradictions of the bank financial capital pool model over the past 20 years cannot be solved overnight. The transformation of the capital management industry must respect the law of industry development and the reasonable elements existing in the present situation. The risk of bank financial management needs to be managed properly. It can be said that if there is no bank to increase the risk preference for customers and bear the risks, there will be no prosperity and development of the capital management market, and customers will not enjoy a relatively high fixed return [19]. At present, bank financing is moving toward strong government supervision. The key to the evaluation of the new regulations depends not only on whether they can properly resolve the problems and existing risks in the capital pool model but also whether the realization of their goals can maintain a moderate balance with the overall benefits to society. Since the financial industry is much more regulated than other industries, government regulation has become a major driving force of industry innovation [20]. Therefore, it is necessary to study the operating mechanism, potential risks, the correlation and coincidence between the capital pool and rigid payment, the complexity of prohibiting the capital pool model and rigid payment, and the legal governance of financial management products. There is also a need to pay attention to the effects of the new regulations on economic operations to strike a balance between preventing systemic risks and promoting the development of financial products. This is also an unavoidable and important research topic in the asset management industry.

The first part of the paper demonstrates the difference and development of the essence of shadow banking and the traditional banks. The paper discusses that the capital pool, maturity mismatch and rigid payment of financial products violate the principle of “managing on behalf of customers” and the principle that the risk should be borne by the buyers of financial products. At the asset end, the money is mostly invested in long-term non-standard debt. These essentially become “savings-like products” or “the shadow of banks”. Risks accumulate in the banks. Curb the disorderly expansion of shadow banking from the source is the most important financial regulatory policy in China. The second part demonstrates the existence value and potential risk of capital pool model in bank financial products. The paper discusses that the capital pool itself is not an illegal concept in the legal sense but is a kind of capital collection form and centralized management mode of funds formed by the principle of “maturity mismatch”, which has existence value and rationality. Therefore, this paper discusses the reasons for banning capital pools in financial products, and analyzes the internal motives and risks behind capital pools from the perspectives of regulatory arbitrage, interest rate policy and deposit competition, and also analyzes its correlation with the rapid expansion of bank financial products. The third part demonstrates the complexity of the supervision and prohibition of the capital pool model in the bank financial products. The capital pool is basically characterized by the result of capital preservation and income protection, and has the integrity of operation. The prohibition of capital pools and rigid payment will affect the development of shadow banking, breaking the close relationship between wealth management funds and the funds in banks' balance sheet. The supervision and governance are difficult and complicated. The fourth part discusses the influence and implementation effect of “The New Regulations on Asset Management”. The “New Regulations on Asset Management” is relatively successful in eliminating the maturity mismatch and preventing systemic risks caused by liquidity problems, and resolves the contradiction between investors' concentrated redemption and issuers' guarantee of capital liquidity by strong intervention. In practice, there are many differences in the understanding of the specific provisions of “The New Regulations on Asset Management” and evaluation of its policy and implementation effect, which also means that there is a probability of marginal relaxation. The fifth part puts forward the suggestions on the supervision of the capital pool in the bank financial products. The paper discusses the existing value and potential risks of China's shadow banking business model, and puts forward relevant policy suggestions for improving the external supervision of shadow banking and optimizing the internal control mechanism of shadow banking. The safe and controllable capital pool model is conducive to improving the allocation efficiency of the financial market, and there is still great room for improvement in “The New Rules on Asset Management”.

Through the analysis, this paper puts forward that the moderate shadow banking scale has a positive effect on macro economy, excessive shadow banking scale will bring negative impact and reduce the financial stability. Therefore, it is necessary to improve the ability of regulation to support economic growth without threatening financial stability. The principle of moderation is needed between the financial security value and the overall interest development. Legislators may need to jump out of the model of prohibitive regulations and establish a principle-oriented regulatory model instead, release more flexibility, maintain a certain elasticity of regulatory policies, and give regulators appropriate discretion, so as to create a modern regulatory system that combines rigidity and flexibility.

To determine whether a financial innovation is worthy of affirmation and encouragement, the essence is to judge whether it can improve the use and circulation efficiency of funds, and to explore whether it can realize the purpose of rational allocation of financial resources, so as to meet the objective needs of the current economic development. The correct direction and focus of supervision should point to the investment behavior of bank financial products in the field of non-standardized debt assets, rather than adopting a one-size-fits-all approach to the capital pool model. It is necessary to evaluate the effectiveness of the regulatory policies already implemented, including whether they have accurately simulated risks from non-bank financing, or whether they miss a new source of critical systemic risk, and whether they have strived to transform shadow banking into resilient market finance.

## 2 The nature of the capital pool of shadow banking and the difference and development with the capital pool of traditional banking system

It is necessary and reasonable for traditional banks to form depositors' capital pool and apply maturity mismatch. A debt relationship is established between customer and bank with rigid payment and fixed interest rate. Bank needs to repay in accordance with the contract, unless the bank goes bankrupt. In this case, banks bear the risk of not recovering the medium and long-term loans to their customer, which would expose them to liquidity and interest rate risk. The essence of financial products is “financial management on behalf of customers”. The risk of the underlying asset should be borne by the customer rather than banks. Banks only charge fixed management fees. The formation of capital pool and maturity mismatch in financial products will inevitably lead to the phenomena that financial products of a specific term cannot correspond with the underlying assets invested accordingly. This denies the customer bearing their own risk. Banks can only return to the traditional rigid payment and promised interest model. The risk would eventually borne by the bank. In addition to the management fees, the difference between the investment income and the promised interest is obtained by the bank. In this mode of operation, financial products are almost equivalent to customer savings, which essentially breaks through the management regulations of the regulatory authorities on medium or long-term loans of banks, and indirectly carries out the marketization of deposit interest.

Commercial banks are subject to strict supervision in terms of risk control, loan interest rate and loan flow direction. In order to avoid regulatory constraints, off-balance sheet asset and liability business has become the replacement and supplement of bank credit business, which is called “the shadow of banks”. Shadow banking only takes place in Chinese banking system, also known as Chinese-style shadow banking [21]. Shadow banking can be described as credit intermediation involving entities and activities outside the regular banking system [22] that carries out activities such credit conversion, maturity conversion and liquidity conversion [23]. China's shadow banking has the characteristics of “banking centralization” [24]. It takes banks as the core and is based on bank's financial products. The asset quality of shadow banking is not subject to effective supervision and constraints. Theoretically, it can expand infinitely. Therefore, it is one of the most important financial regulatory policies at present to curb the disorderly expansion of shadow banking from the source. In order to maintain the balance the capital end and the asset end, shadow banking institutions have to provide short-term loans to meet the long-term capital needs of enterprises through the method of rolling. The maturity of the capital end does not match the asset end, and there is also a mismatch between the term of the bank's investment assets and the liquidity expectation of investors [25]. The maturity mismatch and capital pool model are prominent characteristics of shadow banking. In this mode of operation, multiple financial products correspond to multiple assets. It becomes difficult to identify the asset source of the income of each financial product, and the risk becomes difficult to measure. The short-term financial funds are put into long-term debt or equity projects, which increase the liquidity risk of financial products.

(1) The source and characteristics of capital end of Chinese shadow banking: wealth management products

The reason why wealth management products are regarded as an integral part of the shadow banking system is that they also play the credit intermediary function to replace traditional banks by using the principle of “maturity mismatch”. After 2008, the financial products of commercial banks have experienced a rapid rise and constituted an important cornerstone of the Chinese-style shadow banking system [26]. Shadow banking in European and American countries is mainly based on financial innovation and securitization. It carries out asset securitization through non-bank financial institutions, and its business is generally independent from the balance sheet within the system.^2^ However, China's shadow banking is different from other jurisdictions in funding channels, investment modes, and business structures. It is based on the innovation and breakthrough in financial regulations. Its business is mostly formed in the form of " shadow of the banks” on the balance sheet assets of commercial banks and rely on the existence of commercial banks [27]. Shadow banking and traditional banking system provide services with similar functions, which are credit conversion, maturity conversion and liquidity conversion, but the operating models are quite different. This is because in the shadow banking system, the funds that support credit intermediaries come from wealth management products [28]. Chinese shadow banking model is largely influenced by the government regulatory environment. In China, the interest rate has not been fully marketize, the objectives of monetary policy and the process of financial policy are inconsistent, the rigid payment is difficult to break [29]. Shadow banking originates from the background of the increasing financing demand of the real economy and the realistic situation of the insufficient supply of commercial banks due to monetary control. It is the development of the financial system and the refinement of the financial division. It is also a common way to regulatory arbitrage and avoid the legal restriction of the loan-deposit ratio (75%) [30].

For the small and medium-sized banks, the motives to remake the off-balance sheet asset channel and create shadow banking is because the central bank's loans actually favor those large banks represented by primary dealers in open market operations. The inability of small and medium-sized banks to fairly obtain new liquidity from the central bank thus induces them to compete with large banks in their existing funds by issuing high-interest off-balance-sheet wealth management products [31]. Under China's financial system, commercial banks are the main way to transport the capital to the real economy. However, the excessively stringent capital adequacy requirements directly restrict banks' allocation of new debt liquidity to the traditional in-balance sheet loans. As a result, banks can only create a new off-balance sheet asset allocation channel that is completely parallel to the traditional credit channels. As the deposit interest rate is kept at a low level and due to the high inflation rate in China, the negative interest rate of real deposits occurs, which drives the public to shift funds from bank deposits to financial products in shadow banking system. In this case, the loss of deposit also further limits the formal lending capacity of banks. Banks has to rely more on off-balance-sheet financing [32]. Financial products provide investors (depositors) with investment choices other than deposits and have certain price discovery functions. They also indirectly promote the process of marketization reform of deposit interest rate. As of December 2018, the scale of shadow banking in China reached 54.1 trillion yuan, of which the scale of bank wealth management was 32.1 trillion yuan, accounting for 59% [33]. Financial products has become one of the important tools for banks to realize the release of credit assets.

By controlling the issuance and investment of off-balance sheet financial products, banks can create another parallel investment channel of bank financial products in addition to the traditional credit capital channel, and then transform the in-balance sheet credit constrained by strict capital adequacy ratio into the investment of off-balance sheet financial products with relatively small regulatory pressure. Moreover, China's measures to expand domestic demand and promote economic growth, as well as the deposit competition have significantly increased the scale of shadow banking in China. Small and medium-sized banks have significantly increased their shadow banking business through the issuance of wealth management products [34]. In addition, the greater the regulatory constraints on the loan-to-deposit ratio and capital adequacy ratio faced by banks, the more radical the banks deal with competition through shadow banking business, and the more wealth management products they eventually create [35].

In order to reduce the cost of raising financial products, commercial banks have issued a large number of capital-protected financial products, and they have to pay the corresponding principal and interest as promised at the maturity date. From the perspective of asset allocation, banks have replaced or supplemented the traditional credit channels by creating the off-balance-sheet investment channels for wealth management products [36]. This can cope with the increasingly stringent capital regulatory requirements, but does not establish an effective loss compensation mechanism. The typical feature of shadow banking in China is the market innovation with arbitrage financing as the core, which is the gaming product of financial inhibition [37]. When the capital pool connects with the asset pool, wealth management products evolve into real shadow banks. It has the characteristics of rigid payment or rigid payment expectations, with most products promised to protect the capital or the minimum income [38]. It can be summarized as that in the process of issuing financial products, some financial institutions operate the capital pool of the raised funds through rolling issuance, collective operation and separate pricing. The credit intermediary process of shadow banking is often completed through the capital pool, which is an informal asset securitization activity [39]. According to regulatory rules, wealth management activity belongs to the off-balance sheet business of financial institutions. Financial institutions only charge management fees without bearing investment risks. However, facing the potential investment losses of off-balance-sheet financial products, it is impossible for banks to really require investors to be responsible for their own profits and losses.

In the face of the development of shadow banking, the regulators have made some responds. In January 2014, The State Council of China issued the “Notice on Several Issues concerning Strengthening Shadow Banking Business” (Not Officially disclosed No.107) was regarded as the basic law of China's shadow banking field. For the first time, the document included the capital pool business into the category of shadow banking, in which the wealth management products and trust were not corresponded with each other. The document confirmed that shadow banking is a useful supplement to the traditional banking system. Its purpose is to prevent the risks of shadow banking and guide it to develop healthily [40]. The purpose of regulating bank financial management activities is to avoid the asset management business from becoming a disguised credit business and prevent and control the risks of shadow banking [41]. Regulatory policies directly focus on the core characteristics of Chinese shadow banking system, including explicitly prohibiting relevant financial products from promising the principal and income, breaking rigid payment; restricting the investment of financial management funds in non-standardized assets, and prohibiting the capital pool model. The rationality of this policy lies in the fact that by prohibiting the capital pool model in banks' wealth management business and implementing net value management, the investors will bear the investment risk, which will also break the rigid payment at the same time. This will affect the operation of shadow banking to a large extent. Once the financial institutions have issues such as poor management and insufficient profits, the “capital end” will not be able to redeem the total redemption money of investors, and the capital pool would become difficult to maintain. Under the pressure of intensive regulatory policies, the expansion trend of banks' financial management activities began to be artificially suppressed.

(2) The asset end of Chinese shadow banking: standardized and non-standardized debt

Due to the consolidation of debts, the difficulties of banks' lending and bond financing activities have increased. It has led some Chinese state-owned enterprises to finance through shadow banking that has higher risk and interest rates [42]. In 2010, the China Banking Regulatory Commission (CBRC) issued the “Notice on Matters Related to Regulating the Cooperation between Banks and Trust Finance”, requiring banks to include all bank-trust cooperative financial products into the balance sheet. The regulatory requirement weakens banks' advantage of using wealth management products to make loans. There are a large number of “non-standardized” assets in the investment of capital pool financial products, which lacks for corresponding asset price determination rules [43]. In 2013, China Banking Regulatory Commission issued the “Notice on Issues related to Regulating the Investment and Operation of Financial Services of Commercial Banks, which introduces the concept of “non-standardized debts".^3^ The CBRC requires that the balance of a bank's wealth management funds invested in non-standardized debt assets should not exceed 35% of the bank's wealth management products at any point, or 4% of the total assets disclosed in the bank's audit report of the previous year. This has adjusted the entry threshold and investment direction of investors, and defined the proportion limit of non-standard investment.^4^ According to the “Annual Report of China's Banking Financial Management Market (2020)” released by the Banking Financial Registration and Custody Center, non-standardized debt assets are the second largest category of assets held in financial products after bonds and monetary market instruments, and their lowest annual proportion in the total financial assets is 15.73%, with the highest of 27.49% [44].

Whether there will be problems with bank financial products depends not on the maturity mismatch, but on the quality of the underlying assets invested. In essence, there is no obvious difference between non-standardized debt and traditional credit assets. The non-standardized asset plays the credit supply function in off-balance sheet activities in commercial banks. However, it is separated from supervision and risk control of in-balance sheet activities, which is easy to cause systemic risks. Therefore, the regulation should focus on the investment behavior of bank wealth management products in the field of non-standard assets. By giving investors products that have higher interest rate than savings deposit and risk-free return, banks have realized the rolling issue of financial products, established capital pool, and invested in capital pool mainly made up of non-standardized assets. Although wealth management product contracts usually make it clear that investors bear the investment risk, the operation process determines that this clause cannot be implemented.

If there is a capital pool, there is bound to be maturity mismatch. The investment of non-standardized assets grows rapidly and becomes one of the core allocation directions of bank financial products [45]. Before the implementation of “The New Regulations on Asset Management”, in order to avoid credit policy restrictions, commercial banks usually invest in credit restricted enterprises in the form of “non-standardized asset” to meet the financing needs of some small and medium-sized private enterprises [46]. “The New Regulation on Asset Management” directly divides the assets invested by bank financial management into standardized assets and non-standardized assets [47]. At the end of the transition period of “The New Regulations on Asset Management”, commercial banks can take appropriate arrangements and deal with the stock of non-standard creditor's rights assets that are difficult to be returned due to special reasons with the approval of the regulatory authorities [48]. One of the purposes of “The New Regulations on Asset Management” is to reduce non-standard assets. The allocation of non-standard assets at the asset end is greatly squeezed, the standardized assets such as bonds would become the main assets in the allocation of wealth management funds. Its focus is to improve the ability of net management of standardized asset, strengthen the layout of standardized assets in operation, and promote the conversion of non-standardized asset to standard assets [49]. The purpose of limiting non-standard assets is to control the risk of banks' shadow banking business. However, it is undeniable that non-standard financing can indeed provide certain liquidity support for the real economy, which is an important supplement outside the credit system of the bank on the balance sheet and has strong practical significance [50]. From the perspective of the cost of capital, the regulatory requirements of net value management, capital pool and asset pool governance required in “The New Regulations on Asset Management” are bound to bring the fluctuation of yield. Accustomed to the security brought by investment that preserve capital and income, it will be difficult to let investors accept the net value management. This will lead to the loss of risk-averse investors, limit the raising of wealth management funds, and may increase the cost of capital.

## 3 Existing value and potential risk of the capital pool model in banking financial products

The capital pool itself is not an illegal concept; there is no unified identification standard or norm formed in the supervision process and no authoritative interpretation. According to academic and business practice, the capital pool is a type of fund collection formed by using the principle of maturity mismatch. It uses borrowing shorts and lending to match financing parties to realize the function of credit intermediaries [51]. The capital pool relies on the centralized management of funds, which collects funds from different sources and flows in one place to maintain the basically stable amount of funds in the “pool” [52]. The pools have been prohibited because the risks generated in this model are not truly borne by investors but rather accumulate in the banking system, turning the financing business into a kind of credit business without capital management and risk allocation [53]. If the bank carries out the financial management business using the capital pool model without restraint, the entire financial system may constitute a systemic risk of “Ponzi financing"^5^ [54] because of the correlation and overlap between the capital pool and rigid payment. Bank financial products are the main components of the Chinese shadow banking system. According to scholars, “Chinese mainland's shadow banking system is very complex, it mainly completes credit conversion through financing products. In this process, the capital pool model is usually used” [55]. Important questions thus raised: What are the reasons behind the rapid expansion of banking products? What is the internal driving mechanism and rationality of the generation and development of the capital pool? It is necessary to analyze the motivation and risk behind the capital pool from the perspective of regulatory arbitrage, interest rate policy and deposit competition.

A. The Essence of The Capital Pool Model in Bank Financing: Quasi-Market Interest Rate Competition and Excess Expected Benefits

The capital pool model occurs throughout the financial management business and is regarded as a heavy weapon for rapidly expanding bank financing management. From the perspective of functional attributes, the capital pool is a financial tool to manage liquidity, and the original intention is to achieve short-term fundraising and long-term investing through maturity mismatch. By issuing financial products with an expected yield on the capital side, banks raise funds to form a pool, and the asset ends invest in standardized and non-standardized assets.^6^ In practice, it creates problems such as short-term funds corresponding to long-term assets, multiple funds corresponding to multiple assets, and financial products trading with each other. Given that the capital pool formed by the collective operation can aggregate large-scale funds to concentrate foreign investment, it enhances the flexibility of managers to mobilize funds and helps to improve the efficiency and yield of the use of the funds. This has been adopted by many financial institutions to satisfy investors’ desire to obtain higher returns in the short term and realize financial innovation. As advocated by scholars, financial innovation has changed the entire financial system, and financial innovation is driven by the pursuit of wealth preservation and appreciation [56].

In February 2004, the Shanghai branch of China Everbright Bank issued its first foreign currency wealth management product, “Sunshine Financial A Plan”. In July of the same year, an RMB financial product, “Sunshine Financial B Plan”, was launched. This development signaled that the commercial banks of China had formally entered the asset management industry. Once bank financial products emerge, they quickly become the investment products favored by customers [57]. In 2006, the Industrial and Commercial Bank of China designed a bank financial product that adopted the investment model of creating new shares through a trust. This was the birth of “capital pool-asset pool model” financial products, which connect the capital pool with the asset pool, and capital advantage for arbitrage behavior [58]. In this way, bank financial products, as a form of investment with high yield and low risk, cater to the needs of investors [59]. Through the operation of “capital pool plus expected income (rigid payment)” and taking advantage of the channels of the financial industry, bank financial management has developed to the scale of 30 trillion yuan in just over a decade.

Capital competition in capital markets is the core. The capital competition of bank financial products includes not only different types of banks (large banks and small banks) but also nonbank financial institutions. The Bank of China's loans favor the basic monetary delivery structure policy of large banks, forcing small banks, which are at a disadvantage in incremental liquidity competition, to compete with large banks by issuing high-cost financial products (increasing yield). At the same time, financial products have become an important tool for banks to deal with interest rate marketization and to compete with nonbank financial institutions. As the rate of return or the yield of financial products is not directly controlled by the regulatory authorities, the larger the spread is between the market interest rate and the deposit benchmark interest rate, the greater the number and scale of financial products issued by banks. As scholars believe, the deposit interest rate in the formal financial system is relatively low, and depositors lack a way to invest. These factors induce them to transfer funds from the bank and invest in financial products in the shadow banking system [60]. Bank financial products have formed a counterforce mechanism for deposit and loan interest rates, which has become the forerunner of the breakthrough in interest rate marketization and the driving force of interest rate marketization in China. The requirement of legal reserves and the upper limit of the deposit interest rate strictly limit the profitability of banks [61]. China's commercial banks are subject to strict deposit interest-rate cap controls and balance-sheet lending limits, and issuing financing products has become a strategy for banks to achieve regulatory arbitrage [62]. Bank financing bypasses the limits of the loan-to-deposit ratio, reserves and credit size by releasing “nonstandard” creditor assets. From the perspective of asset allocation, banks can meet increasingly strict capital supervision requirements by replacing traditional credit channels and creating off-balance-sheet investment channels for financial products [63]. With a nontransferable and non-standardized creditor asset, there are problems such as long term, poor liquidity and the existence of credit risk. Commercial banks need to use a collective management approach to managing finance products and “nonstandard” creditors' rights assets. Therefore, the “capital pool-asset pool” model has become the mainstream financial operation model of some banks [64]. In this way, in addition to commissions and fees, the income source of a bank financial business also includes the spread between capital pools and asset pools, that is, the profit model of “management fee or income = asset end income-capital end cost”. According to scholars, commercial banks, as the trustees of funds, externally manage assets in their own name, and there are benefits driven to obtain excess income [65]. Banks essentially treat the funds they raised with financial products as a kind of debt fund. After paying the investor's fixed cost (expected return), all remaining proceeds go to the bank itself.

The capital pool has evolved into a hidden rule in the industry because it has a profound practical rootedness in China's financial environment. The purpose of establishing the capital pool is to gain the ability to actively manipulate the asset pool and manage the liquidity risk caused by the maturity mismatch, which also explains why the maturity mismatch is always associated with the capital pool [66]. In practice, to ensure the continuous insurance of financial products, banks widely adopt rigid payment strategies, which builds more public trust in bank financial products than in other financial instruments [67]. Whether the expected return on financial products is reasonable and whether it corresponds with what is brought by the investment return on investment assets have been ignored by investors. Although the regulatory authorities impose strict constraints and restrictions on financial products of the “capital pool” model, the financial products of the “capital pool” model cleverly evade supervision through portfolio investment and maturity mismatch and have become the common operation model of bank financial products [68]. With the development of financial products and the pursuit of high yields, the capital pool has become the mainstream allocation model of Chinese mainland bank financial management [69]. Many scholars are concerned that one-size-fits-all management kills the capital pool model because the model is only irregulated and does not seek to be a “Ponzi scheme”. The “capital pool-asset pool” operating model has attracted much criticism, with the sharpest model being the “Ponzi scheme”. However, this criticism has not grasped the nature and crux of the problem; it overemphasizes the importance of liquidity. Financial products are a typical short-term debt and long-term maturity mismatch model, and the degree of maturity mismatch is equivalent to that in the traditional commercial banking business. Some scholars even believe that if bank financing is a “Ponzi scheme”, then the whole banking business is a “Ponzi scheme”. The core of the banking business is a maturity mismatch, because even the deposit orientation is not very clear [70]. At present, banking products have not yet entered into the “Ponzi structure”. Relying on its nonnegative spreads and previously retained earnings, “capital pool-assets pool” does not constitute a “Ponzi scheme”. However, if the spread falls further, the risk of causing a “Ponzi scheme” in the future cannot be absolutely excluded [71]. Certainly, the capital pool may only be a transitional model in the development of financial products, and regulators should provide measures for dredging as well as congestion. Positioning the prohibited capital pool practice as a phased means can effectively cooperate with the country to achieve the phased goal of quickly rectifying the financial market as a whole. The capital pool model is not suitable as a long-term means and should not be fixed in the form of a legalization system. It is not appropriate for the capital pool model to be fixed as a long-term means or in the form of a legalization system, but the capital pool model does have existing value and rationality. The ability of financial institutions to carry out capital pool business with maturity mismatch and rigid payment largely depends on their real-time grasp and de facto control of asset profits and cash flow, as well as their investment management ability on the asset side.

B. Malpractice and Potential Systemic Risk of The Capital Pool Model: No Established Effective Loss Compensation Mechanism

Under the capital pool model, the term of the financial products at the capital end is often short, while the term of the investment target at the asset end is longer, forming the maturity mismatch. The maturity mismatch function can only be realized under the “capital pool-asset pool” model. Under the “capital pool-asset pool” model, the operation of funds is opaque, and the risk transfer is insufficient. This maturity mismatch makes the financial return rate not directly linked to the asset profit margin. The income of financial products cannot be accounted for separately, and the risks and income of financial investment cannot be borne and enjoyed by customers, making banks face market risk such that the actual profit margin of assets is lower than the expected yield of financial products. To ensure the sustainable payment of financial products under the fund pool mode, investment income must be higher than payable financial income. In fact, capital pool financial products are mostly supported to invest in some high-risk industries, and their repayment ultimately depends on the return of the asset pool, resulting in financial products facing the risk of a large interest spread. The essence of prohibiting capital pool business is to prevent the mix of funds between bank credit business and capital management business, leading to financial institutions essentially engaging in credit business without meeting the requirements of banking regulatory indicators. According to scholars, financial products operate in a closed internal system of banks, so banks actually undertake hidden guarantees [72]. Unlike ordinary financial risks, once it erupts, the risk of capital pools often evolves into systemic risk [73].

People in the market used to believe that “maturity mismatch” helps to promote long-term investment and meet the liquidity needs of investors at the same time and that it urges banks to maximize the interests of depositors as their business objectives and to maximize their professional skills [74]. However, there are disadvantages of “maturity mismatch”, which magnifies liquidity risk as it accumulates systemic risk in the entire financial system. Maturity mismatch of financial products and nonstandard assets bring liquidity risk [75]. Capital pool financial products operate through rolling distribution and maturity mismatch. Income and risk are mismatched, which weakens the nature of the financial management business and, to some extent, has the attribute of the bank's own business. The largest potential victim of capital pool financial products is the bank itself [76]. If investors do not bear their own risk, then the capital pool financial products may be closer to deposit products. If investors encounter a large number of redemptions, banks must rigidly pay them with their own funds. The capital pool business is closely related to banks' in-balance-sheet funds, and the on-balance-sheet and off-balance sheet businesses are interlinked. Without a corresponding isolation mechanism, the risk may be transmitted to the in-balance-sheet business. Financial products adopting rigid payment are essentially covered by in-balance-sheet funds, which strengthens the degree of correlation between the two. Under pressure, the cost of banks to support financial products may be quite high, and once a run occurs the liquidity of some banks may be under pressure [77]. The operation model of the capital pool is actually to transfer risks between different products and different customers. It does not realize the original intention of matching the risk and income of the financial management business on behalf of clients [78]. Once the risk occurs in the financing project or investment project in the asset pool, no matter how the rigid payment is made, it is replacing the risk with risk and is the displacement of the subject of the risk and return. Some scholars even believe that although the capital pool is an effective credit intermediary, the current mode of operation is very opaque and cannot control its risk or evaluate the quality of assets. This is likely to evolve into a “Ponzi scheme” [79]. There are also professionals who directly believe that the so-called capital pool management business is essentially a “Ponzi scheme” in the form of “rob Peter to pay Paul” and “drum-and-pass” [80]. Because the high returns of capital pool financing cannot ensure that the redemption requirements at maturity of all investors can be met, the capital pool financing business without a risk isolation mechanism can easily endanger the safety of banks.

Prior to the introduction of the new regulations, the average remaining period of financial investment in nonstandard assets was 784 days, while the average remaining period of noncapital preservation products was 129 days. The maturity mismatch was serious [81]. This will inevitably break the principle that the bank's financial products must not trade with each other or adjust their income, which leads to the result that the market risk caused by the maturity mismatch and credit risks caused by rigid payment are both borne by financial institutions. When the financing income of the capital pool and the funds obtained by the new sale of financial products cannot meet investors' due redemption requests, a liquidity crisis will occur. The characteristic of capital pool financial management product operation is a maturity mismatch, so it must continuously roll out the financial management product. Therefore, the capital pool financial management product has greater fundraising pressure and liquidity risk [82]. The income of financial products with rigid payment is a risk-free investment, similar to bank deposits, which results in a large number of risks behind it not being transferred but rather concentrated in the bank's in-balance-sheet financing. In other words, the investment loss of the capital pool has no export and can only be deposited within the bank. The compensation of investment loss can only rely on a small amount of income retained in the capital pool. Long-term accumulation will lead to the capital pool running behind its expense. Once a major loss occurs, it easily leads to the payment of financial products.

Based on the analysis of the operation of the capital pool financial products, the main risks are concentrated on the maturity mismatch between the capital pool and the investment project, as well as the internal transaction and rigid payment for the realization of the maturity mismatch, resulting in a hidden liquidity risk. At present, there is no systemic risk in the capital pool, but regulators have begun to warn of the need to strengthen regulatory regulations. Without strict supervision, the capital pool will certainly produce systemic risks [83]. The reason why the regulators ban capital pool operations is fundamentally due to their ease of causing systemic risk [84]. Therefore, there is large systemic risk in the operation model of the capital pool. Net value financial products have become the mainstream, product investment will be more specific and transparent, and the distribution of product income will be subject to the income brought by real investment assets.

## 4 Complexity of supervision and prohibition of capital pool model in banking financial products

The capital pool has the hidden characteristic of maturity mismatch and rigid payment of investment products, while shadow banking has characteristics such as capital pool operation and off-balance-sheet operation, and it is also the most active field of rigid payment^7^ [85]. Chinese mainland shadow banking usually takes the form of financial products and builds capital pools [86]. Although the capital pool emphasizes collective operation and has a wider scope than rigid payment, it is basically characterized by the result of capital preservation and income preservation. Many financial products are not guaranteed, but banks almost always pay for the principal loss of individual investors [87]. In May 2018, the expected income products with rigid payment characteristics accounted for nearly 90% of the total scale of bank financing [88]. The characteristics reflected by bank financial products have rigid payment or rigid payment expectations, and most products promise to sustain capital or sustain the minimum income [89]. The operation of the capital pool and the transaction between products guarantee rigid payment and have integrity. The prohibition of capital pool and rigid payment will affect the development of shadow banking and break the close relationship between financial funds and banks’ in-balance-sheet financing funds in the past, and it is difficult to supervise and govern.

A. Relevance or Coincidence between Capital Pool Model and Rigid Payment: Bank Implicit Guarantee

The “rigid payment” of bank financial products is a major problem in the development of financial management. It is mainly manifested in the issuance of financial products in the expected yield mode. The actual rate of return of financial products is not related to the result of the “asset pool” operation. Regardless of how the corresponding funds operate and return, and regardless of the corresponding asset risk, banks will pay at the expected rate of return. China's commercial banks are strictly controlled at the upper limit of interest rates, and financial products have become a new business growth point for banks. Once banks are unable to redeem their own financial products, customers will quickly turn to other banks, leading banks to face huge financing pressure. We can say that bank financial products attract investor funds with rigid payment. According to scholars, rigid payment is an economic phenomenon in which implicit guarantees are used to commit investors to the due payment [90]. Once this commitment is established, the nature of the legal relationship in the investment contract becomes a debt relationship, and the financial institution changes from an original trustee to a debtor [91]. There are many explanations for the causes of rigid payment, such as the improper incentive of regulators, the irrationality of investors, the historically excess money supply, and the reputation mechanism of financial institutions, etc. The rigid payment keeps the risk within the financial system [92]. It may transmit the bank's off-balance-sheet business risks to the in-balance sheet business [93]. The liquidity risk of the capital pool caused by the maturity mismatch will be greater than that of ordinary finance, and the risk can even lead to the occurrence of systemic financial risk.

The inertial thinking and practice of rigid payment is not conducive to the long-term healthy development of the asset management industry. In the short term, rigid payment is a rational decision of financial institutions, but in the long run it is an irrational choice [94]. The deep root of rigid payment is China's long-term financial interest rate control, mainly the price control of bank market funds, and the core is the control of the deposit interest rate [95]. Rigid payment is reasonable on the micro level, but there are macro problems, such as keeping risks within the financial system and not conducive to protecting investors' overall and long-term interests [96]. Rigid payment prevents the risk of basic financial assets from passing along with earnings to investors, resulting in the risk of basic assets. In particular, credit risk is mainly retained in banks. In essence, the bank is providing credit endorsements for financial management, and financial management becomes the bank's liability. At present, the financial management of rigid payment is calculated out of banks' off-balance-sheet financing without capital provision, which virtually increases the capital adequacy rate of the bank and implicitly magnifies the systemic risks of the bank. Therefore, rigid payment affects the decisive role of the market in the allocation of financial resources. The credit ability of financial institutions is “abused” in a sense, which poses a certain threat to the net capital of banks and has a great negative effect on the maintenance of the national financial market order.

However, it cannot be denied that rigid payment provides a stable foundation for the development of the financial industry in a specific period. It is the “centrifugal force” of market interest rate control and the rational choice of market subjects under the pressure of price competition. As discussed by scholars, rigid payment rules enhance the trust of financial products, win the trust of investors and promote the rapid development of financial products [97]. The existence of rigid payment has maintained the stability of the financial environment to a certain extent and buffered the transmission of real economic risk to investors under the situation of severe downward pressure in the economy [98]. Maintaining rigid payments ostensibly imposes unnecessary obligations on financial institutions, but it can maintain the external reputation of financial institutions, create the illusion of zero risk and high returns for investors, increase customer stickiness and lay the foundation for financial institutions to further expand their asset management business [99]. The guarantee clause provides a rigid constraint to solve the problem of entrustment cost, which helps to urge the trustee to work diligently and prevent moral hazard [100]. More scholars believe that in terms of China's financial development, allowing commercial banks to provide customers with guaranteed income products is conducive to improving the product structure of commercial banks and in line with the development trend of the personal finance business and market [101]. Rigid payment does not simply have disadvantages. However, there are more disadvantages than advantages in long-term development [102]. It is the inevitable trend to break the rigid payment of asset management business, but we still need to think carefully about how and when to break it. Under the premise of adhering to the bottom line of no systemic risk, breaking the rigid payment should not wait for the asset bubble to burst itself. It is not advisable to forcibly break the rigid payment in the short term. The process of breaking rigid payment too quickly may artificially trigger the credit risk of the position holding subject [103]. At present, the thinking of rigid payment is still rooted in financial institutions, the majority of investors and even the whole financial system, and financial institutions still have a certain ability to defuse the risk of payment. Suddenly, breaking the rigid payment will lead to instability in various aspects and bring significant damage. Therefore, regulatory authorities should weigh the advantages and disadvantages of various methods of breaking rigid payment and leave certain time and space for financial institutions to digest policies and release risks slowly.

Rigid payment and maturity mismatch belong to the traditional operation model of the business in banks' in-balance-sheet financing. Its application to financial products violates the original intention of the buyer's risk responsibility principle. It is an inevitable trend for financial products to return to risk pricing, so rigid payment must be broken. However, the prevention of financial risks should be a long-term and continuous important goal of financial markets that needs to be gradually adjusted from the existing “one-size-fits-all” strong regulatory model to the strict restriction, reasonable guidance regulation model, so that rigid payment can return to the original position of market behavior and accept market adjustment. This may be a more reasonable and appropriate institutional arrangement. From the perspective of maintaining the safety of financial institutions themselves, we can take a limited recognition attitude toward the minimum guarantee clause and balance the interests of the client and the trustee. We can limit the licensing scope and income of financial products with rigid payment commitments to give full play to their strengths and effectively restrain their shortcomings.

B. Defects of Capital Pool Supervision Before The Introduction of “The New Regulation of Asset Management”: Imperfect Regulation and Management Mechanism

The capital pool is essentially a technical means adopted by asset management institutions in product structures to avoid supervision. Due to the relatively relaxed financial policy, the financial product market is growing rapidly, and the capital pool has become one of the important wealth management tools. However, because the corresponding regulatory legal system is not sound enough, defects such as maturity mismatch and inability to independently account are also highlighted. The healthy development of bank financial management cannot be separated from a unified and coordinated regulatory policy. Before the promulgation of “The New Regulations on Asset Management”, the regulatory framework left a blank in functional supervision aspects, which provided a strong motivation for regulatory arbitrage. In the “capital pool-asset pool” structure, the real investment transaction risk was covered up layer by layer. As scholars believe, the capital pool model is essentially the alienation of the credit function, and the “absence” of supervision further disturbs the order of financial development [104]. The core of supervision should be located in the capital pool from the start [105].

China's regulatory management of the capital pool actually began at the time when financial products entered the market. In 2015, the China Banking Regulatory Commission (CBRC) promulgated “The Interim Measures on the Administration of Personal Financial Management of Commercial Banks”,^8^ which stipulates that commercial banks shall not commit to specific customers the available investment income (Article 22) and that the financial funds collected by commercial banks in the sale of financial management plans shall be managed and used in accordance with the provisions of the financial management plans, and special accounts shall be established for independent detailed accounting (Article 32). In practice, these regulatory requirements play a certain role, but this does not solve the fundamental problem. Breaking rigid payment requires strong means of supervision and corresponding punishment measures. No bank is willing to be the first to break rigid payment. Although most financial products do not indicate capital preservation on the specification and contract, rigid payment still exists. If only one bank breaks the rigid payment and other banks do not, it will also lead to that bank being quickly abandoned by the market [106]. By 2011, with the unprecedented development of financial products, the regulatory financial products for banks entered a period of policy standardization and guided the financial business to develop from regulatory arbitrage to a real asset management direction through policies. Regulators focus on aspects in financial management, including credit supervision arbitrage, deposit supervision arbitrage and the disadvantages of the capital pool model [107]. Many regulatory measures have been introduced for financial products, and the capital pool has been banned by regulatory agencies and industry associations. The rectification and regulation of the capital pool has the dual role of preventing rigid payment and maintaining the independence of asset management [108]. Before the promulgation of “The New Regulations on Asset Management”, the regulatory departments formulated various administrative supervision norms and continuously refined and deepened the supervision of the “capital pools” of various financial institutions. Since 2013, there has been an issuance of more than ten consecutive normative documents, which shows the universality and high risk of the capital pool business.

In 2013, the China Securities Industry Association issued “The Circular on Regulating Matters Related to the Cooperation between Securities Companies and Banks in the Management of Targeted Assets” [2013] No. 124. In 2014, the general office of the China Banking Regulatory Commission issued “The Guidance on the Supervision of Banking Financial Management Business in 2014” [2014] No. 39. In 2015, the Securities Investment Fund Association of China issued “The Rules for the Prohibition of the Implementation of the Eight Bottom Lines in Asset Management Business by Securities and Futures Trading Institutions” [2015] No. 44. It is clearly stipulated that financial institutions shall not carry out capital pool business, but there are differences in the execution of prohibited capital pool and rigid payment between classified asset management products under different regulatory bodies. In 2013, China's General Office of the State Council promulgated “The Notice on Strengthening the Supervision of Shadow Banks” [2013] No. 107, which is regarded as the basic law in China's shadow banking field. It stipulates that financial funds in commercial banks managed on behalf of customers should be separated from their own financial funds. They shall not buy bank loans, shall not carry out capital pool business and should effectively ensure that the source of funds and the application are corresponded one by one.^9^

Prior to the promulgation of the new regulations in April 2018, Chinese government departments strengthened the supervision of the capital pool, but there were also many problems. There is a lack of unified legal norms for financial products of the same legal nature, the level of effectiveness of regulations is low, and there is also a lack of substantial and effective regulatory measures and punishment. Financial institutions still have the tendency of arbitrage. Moreover, these norms are mainly aimed at the lack of information disclosure and supervision in capital organizations, which is common in China's asset management industry [109]. Certainly, the absence of information disclosure is the cover of rigid payment and should be managed. Since the capital pool model involves multiple financial industries, the coordination and cooperation of various regulatory departments have become particularly important. According to scholars, the supervision of financial products should be “homogeneous and regular”, and the effectiveness level of norms should be high, not limited to the industry, and can provide an authoritative basis for judicial judgment [110]. For the disadvantages of arbitrage space generated by standard differences under industry regulation, it is necessary to regulate the capital management business by strengthening regulatory coordination and macroprudential management, as well as implementing functional supervision in accordance with the principle of “substance is more important than form” [111]. Only for the essential characteristics of the capital pool are there some differences in the regulatory documents. Some attempt to define the capital pool business from the behavior results^10^, and some directly adopt the perspective of prohibited operation and clarify what behavior may constitute the capital pool business [112]. Of course, more regulatory documents start from the operation mode and strive to accurately grasp the essential characteristics of the capital pool business.^11^

Commercial banks take capital pool financial management as an off-balance-sheet business and are not restricted by regulatory restrictions such as deposit-to-deposit-loan ratio, which trigger maturity mismatch and rigid payment, violate the firewall system of self-owned businesses and financial management businesses, and easily form systemic risks. Government regulatory authorities have issued a number of documents to regulate bank capital pool financial management business. However, these regulatory requirements have failed to substantially change the capital pool operation model of bank financial products [113]. In practice, problems such as related transactions of capital pool financial products and externalization of the double balance of assets and liabilities are common, and it is difficult for financial regulations to effectively regulate the non-compliance of capital pool financial products. However, the government's core concept of risk prevention has not changed, especially since the strict regulation of bank financial management has not been relaxed [114]. Regulations on the capital pool did not eliminate this business model because the capital pool business is naturally the basis of bank financial management. Whether the overall liquidity of financial business can be guaranteed and achieve sustainable growth has much to do with the operation of the capital pool. The contradictions of “high income” and “high liquidity” inevitably produce the “capital pool” business model.

## 5 Influence and implications of “The New Regulation of Asset Management” in practice

“The New Regulation on Asset Management”, which was implemented in April 2018, has formulated unified regulatory standards for similar asset management products issued by financial institutions, and has made relevant provisions on the implementation of penetration management, strengthening risk isolation, breaking rigid payment, disclosing full information and revealing risks. With the joint efforts of financial management departments and financial institutions, the capital management business is transformed in an orderly manner, and the capital management products have gradually returned to their original source. The proportion of net worth products has increased steadily. By the end of 2019, the balance of funds raised by net worth asset management products was 43.9 trillion yuan, accounting for 55.2% of all asset management products, with an increase of 8.8% year-on-year [115]. By the end of 2020, net worth products accounted for 67.28% of the remaining balance of bank wealth management products, with an increase of 22% [116]. In more than two years of practice, “The New Regulations on Asset Management” has been relatively successful in eliminating maturity mismatch and preventing systemic risk caused by liquidity problems. It has resolved the contradiction between investors' concentrated redemption and the issuer to ensure capital liquidity by strong intervention and will also reshape the development trend and pattern of the capital management market. Of course, in practice, there are differences between the understanding of the specific provisions of “The New Regulations on Asset Management”, the evaluation of the policy effect and the implementation effect, which also means that there is a probability of marginal relaxation.

A. Complexity of Banning the Capital Pool and Rigid Payment Model: Presence Of Rational Value and Positive Factors

The promulgation of “The New Regulations on Asset Management” has opened the era of strict supervision of the capital management industry. It explicitly prohibited the capital pool business model and put forward the “three separate” (separate management, separate setting up account, separate accounting) management requirements. The introduction of net value management makes investment operations more transparent and ensures the rationality and accuracy of the valuation and pricing of asset management products (according to the principle of fair value), which will force banks to adjust the issuance ideas of existing financial products. As scholars have concluded, the core content of “The New Regulation on Asset Management” is to break the rigid payment, standardize the capital pool, remove multilayer nesting, restrain the ratio of leverage and guide the net worth transformation of bank financial products of nearly 30 trillion yuan [117]. The aim is to encourage the asset management business to return to the business source of “entrusted and on behalf of financial management”. The risks of financial products are borne by investors, and the income between products is no longer adjusted by related transactions. However, from the specific rules, many reflect not innovation but the expansion and application of relevant regulatory measures from the China Securities Regulatory Commission, issued in the field of big asset management. “The New Regulations on Asset Management” require banning the publicity of “expected returns”, making banks lose the two gold signs of expected returns and rigid payment [118]. Under the guidance “The New Regulations on Asset Management”, “The commercial bank financial business supervision and management measures”^12^ and “The commercial bank financial subsidiary management measures “^13^ have released, a series of new management policies also encourage banks to adopt a subsidiary capital management business. Bank financial subsidiaries become a beneficial institutional arrangement, with the reform direction of “net worth, marketization, corporatization” on bank capital management business formally established to reduce or eliminate maturity mismatch and liquidity risk on the capital sources end and asset end, put an end to possible systemic risk and make net worth transformation become the general trend.

In practice, the concepts of open operation business and fund pool business are often confused; both are fund collective operations, but the connotation is completely inconsistent. The open operation allowed in “The Securities Investment Fund Act” also essentially contains the core meaning, that is, the net worth management.^14^ For the capital pool business, the prices of subscription and redemption take the method of separate pricing and are divorced from the actual value of the assets. The most essential difference between capital pool business and open operation business is whether net worth management is adopted. If net worth management is adopted, then it is fully in line with the regulatory provisions of the open operation business; if separate pricing is adopted, then that is the capital pool business prohibited by the regulatory authorities. This is also the fundamental intention of why “The New Regulations on Asset Management” emphasize that “institutions should implement net worth management of asset management products”. If there is a capital pool, there is bound to be a maturity mismatch. The restrictions on maturity mismatch in “The New Regulations on Asset Management” are limited to nonstandard assets, and the standardized assets for public trading are not listed in the control of maturity mismatch.

Article 19 of “The New Regulations on Asset Management” clearly stipulates that financial products should not be paid rigidly, which has attracted high attention from all walks of society. As a powerful means to prevent systemic risk in the financial market, the significance of breaking the rigid payment is undeniable. The first civil judgment of “invalid rigid payment commitment” appeared in the Hunan Provincial Higher People's Court in China.^15^ As discussed by scholars, the net worth of financial products, breaking the rigid payment and returning to the source of off-balance-sheet business can help to reduce the systemic risks of the banking industry in the medium and long term and is conducive to its stable operation [119]. However, it must also be recognized that although the behavior of rigid payment increases the risk of financial institutions, it is reasonable for it to exist. To some extent, rigid payment is the result of the combination of various factors, such as the game between developing speed and quality of asset management business, the game between financial regulators' preference for risk control and maintaining financial stability, the game of profit and loss of financial institutions, and the game between investors' savings and investment thinking [120].

Rigid payment is of positive significance in protecting investors in financial products and promoting the development of financial products [121]. The “asset pool-capital pool” operation model in bank financial products has low risk and stable returns, and the risks of the whole industry are generally controllable, which plays a positive and important role in the development of asset management [122]. The adoption of the “one-size-fits-all” regulation method of prohibiting rigid payment ignores the reasonable factors existing in practice. Although it can effectively reduce the systemic risks of financial markets in a short time, it is not a complete plan. The huge volume of bank financial products in the market has been used for the financial models of “income determination before advance” and “rigid payment after event” for a long time. It is incredibly difficult to break the rigid payment simply according to the relevant provisions of the regulatory documents. Under the regulatory requirements prohibiting the operation of the asset pool, a maturity mismatch will no longer exist, which will greatly test the overall operation level of bank asset management [123].

At present, supervision is actually a dilemma. On the one hand, only when investors realize the risks of investing in financial products can the financial products market develop normally. On the other hand, breaking the rigid payment may lead to unnecessary panic and even systemic risks in real time. It is truly challenging to find a balance between these two ends, but in the long run the goal is that buyers should bear their own risks, and this may involve a series of trial-and-error processes. The reason why “The New Regulations on Asset Management” prohibits rigid payment is very clear, which is to prevent systemic risks in the financial market. Credit intermediary activity in the asset management industry is contrary to the spirit of supervision [124]. However, if such systemic risk can be prevented, then the necessity to prohibit the rigid payment of financial products will be greatly reduced. Therefore, it is necessary to rationally examine the rigid payment of financial products again. Rigid payment is like a double-edged sword, and its impact on bank financial management is complex. On the one hand, it is damaging, and the risk of maturity mismatch impacts the order of the financing industry. On the other hand, it also plays a role in building structure. It maintains financial stability, improves institutional reputation and promotes the due diligence of managers [125].

The main cause of rigid payment lies in inadequate and unbalanced financial supply as well as non-marketization. To control rigid payment, it cannot be completed by the formulation of regulations. It largely depends on the improvement of risk preference at the capital end, and it inevitably needs a process to rebuild the risk consciousness and investment concept of customers. The yield of bank financial products is regarded theoretically as a “quasi-market-oriented deposit interest rate” [126]. If we do not reform some fundamental causes and problems, the so-called breaking of rigid payment, or even the breaking of rigid payment through regulatory norms, is poles apart [127]. Similarly, the operation of capital pool financial products has a long history and will not completely disappear simply because of supervision measures. After all, this is an innovation brought by market demand. It not only meets the needs of investment and financing but also allows financial institutions to actively manage assets and triggers a huge market demand. Therefore, because of the rationality and existing value of the capital pool itself, the one-size-fits-all regulation method cannot truly solve all the problems but simply pushes the problems to concealment. Setting up accounts independently and independent accounting may form a superficial phenomenon. Banks make all products share all investment returns and risks through substantive transactions between financial products, and all products are combined to form a capital pool. From the current perspective of bank financial management operations, truly achieving one-to-one correspondence and completely banning the capital pool will produce liquidity problems and may lead to the deterioration of the overall balance sheet of the banking industry, which is not the result that regulators are willing to see. Therefore, the capital pool business cannot be thoroughly checked and cleared in a short time. Instead of completely blocking the capital pool model, it is better to improve relevant regulations on the basis of the existing system, make banks’ financial products mature and standardized as soon as possible and allow moderate innovation when risks are controllable. The responsibility of the regulatory authorities is to control the relevant risks at a single appropriate level, starting from the principle of protecting the interests of investors and maintaining the steady operation of commercial banks.

B. Effectiveness of Supervision and Measures of “The New Regulations on Asset Management”: Balance between Safety Value and The Development of Overall Interests

“The New Regulations on Asset Management” aims to uniformly regulate the asset management business of various financial institutions, implement fair market access rules and supervision, eliminate regulatory arbitrage and realize the classified supervision of the fundraising end and the investment end. Compared with those previous regulations, “The New Regulations on Asset Management “puts forward systematic regulatory measures and increased punishment intensity. In the short term, the development speed and scale expansion of the asset management business will bring certain constraints, which are also the inevitable industry pain brought by the transformation of the product structure and investment structure. The proportion of standardized assets in asset allocation may continue to increase, nonstandard investment is limited and net worth transformation is difficult. Limited nonstandard asset investment will directly lead to difficulty for bank asset management to give full play to their inherent advantages, and the overall income of the financial portfolio may also be reduced. Bank financial management and investment management ability have become the core of solving problems [128]. In the long run, “The New Regulations on Asset Management” will fundamentally correct the illegal operation of the asset management industry and promote the asset management industry to gradually turn to standardized development. As mentioned by scholars, eliminating the phenomenon of maturity mismatch from the source solves the contradiction between the concentrated redemption of investors and assurance to capital liquidity of investors and curbs the occurrence of “risk resonance” to a certain extent [129].

Since their birth, China's bank financial products have been positioned as alternative products of high-interest deposits to a certain extent, and accordingly they have gradually formed a market pattern dominated by capital preservation financial products and expected income products with the characteristics of rigid payment [130]. The focus of the dispute over income-based financial products is not whether commercial banks should provide such products and services, it's the possibility of commercial banks transforming such products into quasi-savings deposits, into a tool for high-interest storage and scale expansion to break through the control of the national interest rate and carry out unfair competitions [131]. The capital pool financial management model is widely used in the field of asset allocation, which involves a wide range of industries and fields, and supervision has become complicated. These also put forward higher requirements for the ability of the regulatory authorities. It is necessary to strengthen regulatory experience and regulatory technology and effectively transform institutional supervision to functional supervision to ensure the flexibility and appropriateness of supervision. Regulatory authorities need to control the relationship between risk prevention and steady growth and promote the transformation and development of the asset management industry in an orderly manner. Before the implementation of “The New Regulations on Asset Management”, commercial banks usually invested financial funds in credit restriction enterprises in non-standard forms to avoid credit policy restrictions [132]. Financial investment in the “non-standard form” is the best way to support and serve the real economy, with high investment and financing efficiency and less additional cost of enterprise financing. The investment income of the “nonstandard form” is stable, which is conducive to financial products. “Non-standard form” bank financial investment belongs to the performance of differentiated competition with funds. Therefore, it is correct to focus on the supervision direction of investment in bank financial products in the field of non-standardized creditors' rights assets, but a one-size-fits-all approach should not be an approach to the capital pool model. Whether bank financial products will go wrong does not depend on the maturity mismatch, but it depends on the quality of the underlying assets invested, which is the core. “The risks from shadow banking and financial intermediation in general crucially depend on the quality of the underlying assets” [133]. The focus of bank financial product operations is to check the underlying assets. Even the commonly existing capital pool model is also invested in trust, claims and other low-risk assets in general. As long as banks continue to adhere to this low-risk and stable investment style, including bank financial products operated by the capital pool mode, there will not be systemic risk [134]. Reasonable and compliant investment of financial products in nonstandard asset businesses is of great significance for the real economy. We should prevent the significant adverse effects of sudden overregulation. A clear macroprudential approach will be key to ensuring that market-based finance continues to meet the needs of society [135].

The implementation of “The New Regulations on Asset Management” has caused a direct impact on off-balance-sheet financing, and the financing channels led by asset management products are difficult to sustain, which seriously weakens the previous financing of small and medium-sized private enterprises. The financing market presents a shortage of supply. After the introduction of “The New Regulations on Asset Management”, the scale of shadow banking financing shrank rapidly [136]. From the current practice, after investors have been used to the concept of “bank financing = high return time deposit” and treat bank financing as a substitute to deposit to enhance income, it is clear that there are no guarantees for high returns or even capital preservation, which will have a huge impact on the capital side. The attraction of investors will undoubtedly decline and have a negative impact on banking financial business. The net value transformation of the existing financial products is also facing great pressure. China's banking industry is undergoing transformation, the profit model with deposit and loan spreads as the main source of profit will gradually weaken, and the off-balance-sheet financial business as a source of noninterest income plays an important role in bank profit composition. When regulators claim to suppress financial innovation with security value to maintain financial order, they are mostly trying to reduce risks, but the pursuit of security value cannot be developed independently of the overall interests of the financial market. As scholars believe, it should be advocated to guide the reasonable development of the asset management market with moderate rules, abandon the excessive constraints caused by strict supervision and release a certain innovation space to protect the richness and diversity of the financial market [137]. If “The New Regulations on Asset Management” is not only conducive to improving the inefficient and rigid financial order, leading it to the fair distribution of financial resources, but also does not damage the situation of financial security and produces more social benefits, that means that the regulation contains legitimacy and rationality. Although “The New Regulations on Asset Management” prevents the occurrence of systemic financial risks by banning capital pool, breaking rigid payment and clearing maturity mismatch measures, at the same time there is no obvious promotion of overall market development, or there are strong inhibition and obstacles, and its realistic guidance and value are worth discussing because promoting the security and stable development of the financial field is the purpose of supervision. The “risk-yield” preference of the majority of investors has not been well explored, and the new asset management system in line with the requirements of “The New Regulations on Asset Management” needs to grow together with the majority of ordinary investors.

There are also several specific problems that need to be further improved and clarified in “The New Regulations on Asset Management”. First, the unified standard of asset management products needs to be further cleared and defined; second, it is necessary to formulate more scientific valuation methods to calculate the product net value; third, the duration management of some products needs to be further improved; and fourth, the definition of open products is not clear enough. More importantly, the focus of departmental normative documents is not on the interest protection of investors but on maintaining the risk control of financial institutions and the systematic security of the financial market [138]. Investors' rights and. The protection of their interests is not a priority; although the regulatory documents never deny the significance of investors' protection, their primary purpose is obviously to ensure the effectiveness of risk management. The rights of investors and the obligations and responsibilities of the trustee have not received clear and complete legal confirmation in “The New Regulations on Asset Management”, and the inclusion of investor protection policies in the regulatory framework is helpful to strengthen financial stability. The purpose of unifying regulatory standards in the asset management industry is to bridge regulatory gaps, eliminate regulatory arbitrage and prevent the occurrence of financial risks. Judging from the implementation effect of the regulatory model, strict regulation may not necessarily eliminate the negative impact of arbitrage innovation on the financial market. The substantive problem that the capital management industry currently faces is which regulatory system can more properly handle the dynamic game between financial innovation and supervision. A constantly evolving and innovating financial system is a hallmark of a functioning market economy [139]. The promulgation of “The New Regulations on Asset Management” and many previous regulatory regulations shows that financial innovation and financial supervision are in a state of “one man's loss is another's gain.” The best choice for regulation is to address financial innovation with a sustained, principle-oriented and risk-based regulatory approach.

## 6 Suggestions on the supervision of capital pools in bank financial products

Under the capital pool model, the final cash flow of financial products is not clear, which, to some extent, covers up the quality risk of assets, increases the difficulty of risk control, and fails to let the investors' bear their own risk on the basis of information disclosure. Therefore, banks must implement the method of rigid payment for wealth management products. The bank guarantees and covers the risks of investment, which violates the provisions that the bank's shall not use the credit funds to provide guarantee for the bank's financial products. Any investment income beyond the expected return rate also belongs to the bank, which makes the capital pool financial products deviate from the original intention, which is financial management on behalf of customers. The factors that shadow banking may cause systematic risk include: maturity mismatch, liquidity conversion, credit conversion and high leverage. Strengthening the supervision standard of shadow banking is an important subject for the global financial industry. If the shadow banking regulation is insufficient, it may induce banks to engage in shadow banking activities and lead to the issue of regulatory arbitrage. At the same time, it is also necessary to explore the existing value and potential risks of China's shadow banking business model, so as to put forward relevant policy suggestions for improving the external supervision and optimizing the internal control mechanism of shadow banking.

(1) The risks and defects of Chinese-style shadow banking and the beneficial supplement to the financing system in financial market

As a financing intermediary, shadow banking business has the functions of maturity mismatch and liquidity conversion, which leads to the high liquidity risk, and it is difficult to measure the basis of the risk. Although intermediating credit through non-bank channels can have advantages, such channels can also become a source of systemic risk, especially when their interconnectedness with the regular banking system is strong [140]. Systemic risks can arise not only from shadow banking entities but also from connections between banks and shadow banking entities. “Banks and shadow banking entities are highly interconnected, with banks often being part of the shadow banking credit intermediation chain or providing (explicit or implicit) support, such as guarantees, to the shadow banking entities to enable cheap financing and maturity or liquidity transformation” [141], or even be owners of shadow banking entities. Therefore, liquidity crisis in a shadow banking entity (or a bank) may bring risk contagion and risk resonance effect to a bank (or a shadow banking entity) [142].

Most of the shadow banks in China are the replacement of bank loans. The customer rating standard is significantly lower than that of loan customers. Both the financing source and the capital investment bear direct credit risks, and the risk is greater than that of bank loans. In terms of withdrawal, the risk mainly arises from the short-term wealth management products. The sources of bank financial funds are mainly from ordinary individual investors. The proportion is basically over 60%. In 2018, it reached 86.9%, and the risk of centralized payment is relatively large [143]. Due to the off-balance sheet operation mode of wealth management funds, the effective process and risk control mechanism of traditional credit asset, including filtering, issuance and supervision cannot be directly applied to the allocation process of off-balance sheet wealth management funds. By collecting different products funds into the pool, neither regulators nor investors can accurately and timely understand the return and risk exposure of individual asset management product, which is not conducive to protect investors from the micro level, and is even less conducive to grasp risk changes from the macro-prudential perspective and prevent systemic financial risk. Also, the capital return requirements of financial products are too high, the cash flow stability is relatively poor, the use of funds does not match the risk bearing, and the risk prevention and protection mechanism is insufficient [144]. The liabilities and assets in a large number of capital pools and asset pools cannot be identified accordingly, which results in serious liquidity mismatch. If the subsequent funds cannot be raised in time, the capital chain will break immediately, the liquidity risk will spread rapidly in the whole industry in a short period of time [145]. The regulatory arbitrage of shadow banking not only reduces the effectiveness of the government's financial supervision, but also exposes the whole financial system to greater risks [146].

The capital pool model continuously raises funds through rolling insurance, maintains the balance between the source of financial funds and the operation of financial funds by using dynamic management model, and carries out maturity conversion and liquidity conversion with the mode of “borrowing short-term funds to invest in long-term investment”. It can be said that the original intention of this model is good. However, with the growth of scale, the risks caused by the maturity mismatch, rigid payment, and the inconsistency between products and assets results in concerns about the potential risks of financial products in the operation mode of capital pool. Once the banks give up the operation mode of capital pool and return to the traditional “one-to-one” products, it is bound to lead to the differentiation of risks and benefits of various products. In addition, the asset pool model business intensifies the risk of off-balance-sheet wealth management asset allocation, making its overall risk higher than that of the traditional bank credit projects. If banks give up rigid payment for their financial products, individual risk events are likely to cause a systemic crisis similar to the diffusion mechanism of bank runs.

In fact, China's shadow banking is a replacement and supplement to the traditional credit channels of banks. It provides important economic functions, especially in providing different savings tools and credit to private enterprises that cannot obtain normal bank credit [147]. The vast majority of shadow banking services focuses on the capital demanders outside the service scope of traditional commercial banks, especially the small, medium enterprises. Enterprises with strong financing constraints are difficult to integrate funds from the outside. However, shadow banking business has the characteristics of capital pool operation and off-balance sheet operation, and has a high-risk-preference, which greatly enriches the financing channels of such enterprises and helps to alleviate their financing difficulties [148]. Shadow banking actually combines capital suppliers and demanders, provides solutions for excess funds, plays the effect of redistribution, and actually helps to improve the efficiency of capital distribution and use in China [149]. From this perspective, by acting as a credit intermediary, Chinese-style shadow banking breaks the amount exclusion and resolves the negative impact brought by China's financial repression. It can be said that its existence has reasonable and practical significance [150].

After the introduction of “The New regulations on asset Management”, the financing scale of shadow banking has shrunk rapidly, which breaking the original balance between supply and demand of funds. Under the circumstance that banks' willingness to lend to small and medium-sized enterprises is still low, the shrinking scale of shadow banking limits the capital source of enterprises, reduces the capital supply of those enterprises with financing constraints, and weakens its supplementary role for formal credit services. The shrinking of the shadow banking has an impact on the financing costs, financing constraints and debt maturity structure of the enterprises relying on its financing [151]. Therefore, the one size fits all regulation method would not truly solve all the problems but simply pushes the problems to concealment. The non-standard investment generated based on the traditional credit business of commercial banks will still be an important asset allocation direction and characteristic advantages of bank financing after solving the two difficulties, which are the maturity mismatch and breaking the rigid payment [152]. Under the current financial background of China, shadow banking is the product of long-term financial repression, which can objectively provide financing services for the real economy and support economic growth. As a product of financial innovation, shadow banking has broken the financing shackles of the traditional financial system. The healthy and controllable scale of shadow banking is conducive to improving the allocation efficiency of the financial market [153]. The core issue is how to regulate and guide the healthy development of China's shadow banking under the premise of controlling financial risks and ensuring financial stability, so as to help shadow banking become a beneficial supplement to the financial market financing system.

[2] Supervision on the heterogeneity of Chinese shadow banking: rationality, inevitability and appropriateness of supervision

The essence of the capital pool business is the precipitation and mixing of a large amount of funds in the process of setting up account, accounting and custody of different financial products after the financial institutions raise funds and before investment. China's shadow banking has accumulated for a long time. It has large stock risk. Implicit guarantees and “rigid payment” have not been in fact broken. Shadow banking comes from the arbitrage behavior of banks under different regulatory rules in pursuit of high profits, which increases the infectivity of financial risks and obviously goes against the macro-prudential principle of financial regulation. The pressure of bank's performance and the external financial development level are the important factors that determine the scale of the shadow banking business [154]. Given that the shadow banking phenomenon involves multiple financial industries, the coordination and cooperation of various regulatory authorities has also become unprecedented. Regulators need to provide a broader platform and more policy support for the standardized transformation of non-standardized products [155].

The empirical evidence shows that the larger the gap between the actual capital adequacy ratio of banks and the target adequacy ratio required by the regulator, the more financial products created by banks, so as to avoid the credit regulatory constraints of the loan-to-deposit ratio and capital adequacy ratio. Non-bank financing can provide a valuable alternative to bank funding. It is also a welcome source of diversification of credit supply from the banking system [156]. Although intermediary credit through non-bank channels has advantages, such channels may also become a source of systemic risk, especially when they are highly interconnected with the formal banking system. Therefore, appropriate monitoring and regulatory frameworks needs to be in place for the shadow banking system. If shadow banking shrinks sharply, borrowers relying on the channel (such as small and medium-sized enterprises) may not get financing even if they still have good credit quality [157]. Although China has regulated the shadow banking system over many years, the attitude of the regulators was not to completely curb the growth of shadow banking. This is because shadow banking has a positive influence on China's industries which capital is in urgent need [158]. Therefore, the shadow banking should be supervised in principle, that is, to encourage the development of shadow banking and bring shadow banking into the scope of supervision. The goal is to leave room for the financial industry to achieve self-discipline and to maintain a balance between efficiency and risk. Since the implementation of “The New Regulations on Asset Management”, the People's Bank of China has, together with the regulatory authorities, guided the benign development of asset management business through measures such as channel removal, rigid payment breaking, mismatch restriction and deleveraging, so as to effectively govern the disorderly expansion of shadow banks [159]. China's regulators have taken steps to eliminate the risks associated with the interconnected banking and shadow banking sectors, but vulnerability remains high, and further regulatory actions are crucial to continue to reduce risk in the financial sector [160]. It is necessary to resolve the stock of non-standard assets through compliance with the new product undertaking, early exit, converting non-standard to standard asset, returning asset to balance sheet and other special treatment methods [161]. This process is complex, and how to dispose of the stock of assets remains difficult. Banks and financial institutions still need certain time and space to digest policies and release risk slowly.

It is expected that in the future, the size of Chinese shadow banking will gradually decline and be controlled, and it will be a competition relying on risk pricing ability and active asset management ability. The possibility of a systemic financial risk caused by shadow banking is very small. The disposal strategy of shadow banking should be to reasonably regulate its development, rather than strictly restrain and suppress it [162]. It is expected that both the demand side and the supply side of capital are constantly innovating, and the market operation and supervision of shadow banking are constantly evolving in the process of mutual game. The regulatory authorities only need to ensure the effectiveness of the financial market. If they blindly restrict and squeeze financial innovations such as asset management products, it will destroy the existing channels to repair financial repression and financial exclusion. This would cause a new round of contradiction between supply and demand of investment and financing. For the market behavior to return to the original position and accept market adjustment, it needs a more reasonable and appropriate institutional arrangement, and inevitably needs a process to rebuild the risk consciousness and investment concept of customers. When trying to weaken the risk and maintain financial order, regulations are generally suppressing financial innovation. However, Pursuing safety value cannot be independent from the overall interest development of the financial market.

As a result, there should be moderate rules to guide the reasonable development of financial market. This includes transforming shadow banking into resilient market-based finance to support sustainable economic growth [163]. A constantly evolving and innovating financial system is a hallmark of a functioning market economy. A clear macroprudential approach will be key to ensuring that market-based finance continues to meet the needs of society. The resilience of nonbanks should be promoted through the development of a range of policies to address systemic vulnerabilities where they arise, while not impeding the growth of sustainable nonbank financing models.

## 7 Conclusion

The continuous development and innovation of the financial system is the symbol of the operation of the market economy. Flexible market finance can complement banking finance in many ways. In fact, those small/medium enterprises’ financing activities provide an example of how these different parts of the financial system can work together to fund critical economic activity. The moderate scale of shadow banking plays a positive role in the macro economy, but the excessive expansion of shadow banking will bring negative effects and reduce financial stability. Therefore, it is necessary to improve the ability of regulations to support economic growth without threatening financial stability. The principle of moderation is needed between the development of financial security value and the development of overall interests. Standardizing and guiding the development of shadow banking in China and improving the modern financial system are of guiding significance for promoting the coordinated development of shadow banking and macro economy.

Bank financial products directly enter the real economy through the reasonable allocation of various assets, which is conducive to the development of direct financing in China, providing diversified investment channels for investors and bringing considerable economic returns. The supervision measure of “The New Regulations on Asset Management” is a realistic choice under the existing industry supervision and bank hidden guarantee system. Strengthening the necessary supervision is the only way to promote the healthy development of bank financial products, but it also has a direct impact on bank off-balance-sheet financing. The most prominent focus is restricting investment in non-standardized assets. Of course, it will also encourage the market to seek a path to avoid these regulations and greatly improve the incentive for innovation. The purpose of “The New Regulations on Asset Management” is to strictly control risks, which is a containment process for the asset management business, and the market will deal with them through innovative means and models.

Compliance supervision of capital pool financial products cannot be achieved overnight. The improvement of the regulatory system should be combined with China's financial supervision system and the financial environment. The root cause of the long-term defects in the capital pool structure is the setting of financial stability standards, which requires a more reasonable risk transmission and sharing mechanism. The safe and controllable capital pool model is conducive to improving the allocation efficiency of the financial market, for which there is still much room for improvement in “The New Regulations on Asset Management”. The core factor of the liquidity crisis possibly caused by the capital pool is how to effectively guarantee the solvency of asset-end debt. The quality of the original “native assets” must be strictly assessed and reviewed, which is the core element of financial innovation to support the development of the real economy, and tests the real asset management ability of asset management institutions.

Bank financial products will break rigid payment and prohibit trading between products, and risks will be passed on to investors with income. Banks can no longer “cover the bottom” for investors. As asset managers, banks assume the responsibility of entrusted management, and the allocation of asset ends of financial products is more difficult, which will transform from paying attention to credit risk management to comprehensive risk management. The net worth transformation of financial products still has a long way to go, and the term of financial products will change from short-term to long-term.

Because of the increasing complexity of the financial market caused by financial innovation, to solve the time lag between law and financial innovation, legislators may have to forsake the main model of prohibition, abandon the traditional and comprehensive rule-oriented approach, establish a principle-oriented regulatory model, provide more flexibility, maintain certain flexibility in regulatory policies, give regulators appropriate discretion and create a modern regulatory system that combines rigidity and flexibility. Whether a financial innovation law is worth affirmation and encouragement is essentially based on judging whether it can improve the use and circulation efficiency of funds and whether it can achieve the purpose of the rational allocation of financial resources to meet the objective needs of current economic development.

## Author contribution statement

Yilin Hu: Conceived and designed the experiments; Performed the experiments; Analyzed and interpreted the data; Contributed reagents, materials, analysis tools or data; Wrote the paper.

## Data availability statement

No data was used for the research described in the article.

## Declaration of competing interest

The authors declare that they have no known competing financial interests or personal relationships that could have appeared to influence the work reported in this paper.

## Footnotes

1 Shadow banking in China mainly refers to the financial commodities issued by banks, or entrusted loans, trust loans, etc. (See M.Wu, Status of Chinese mainland financial risk and Future Development, Economic Foresight (07) July 2020, Issue 07, pp. 64.

2 The Financial Stability Board (FSB) under the Group of 20 (G20) defines shadow banking as a credit intermediary system that is outside the banking regulatory system and may cause problems such as systemic risks and regulatory arbitrage.

3 Non-standardized debt assets are defined as debt assets that are not traded in the interbank market and the stock exchange market.

4 “The New Regulation on Asset Management” stipulates that public offering products should mainly invest in standardized assets, and clearly stipulates the investment proportion. The proportion of all financial products invested in non-standard debt assets still follows the proportion of the “Notice on Regulating the Investment Operation of Financial Services of Commercial Banks”.

5 “Ponzi financing” is often associated with marginal or fraudulent financing activities. The term usually refers to the program that uses the principal of the funds invested by investors entering the program to fulfill their commitment to excessive returns to investors entering the program earlier. “Ponzi financing” is not necessarily fraud, but the growth of Ponzi financing is a sign of a slide into a fragile financial system.

6 “The New Regulations on Asset Management” defines the non-standardized assets, but it is different from the logic previously enumerated by the CBRC and the CSRC. It gives the scope of standardized claims, except for standardized assets, all other assets are nonstandardized debt assets. Standardized debt assets shall meet the following conditions: [1]. Equally divided and tradeable [2]. Full disclosure of information [3]. Centralized registration, independent custody [4] Fair pricing, perfect flow mechanism [5]. Trading in the interbank market, the stock exchange market and other trading markets agreed by the State Council.

7 Shadow banking in China mainly refers to the financial commodities issued by banks, or entrusted loans, trust loans and so on.

8 China Banking Regulatory Commission Order No. 2 of 2005 “Interim Measures for the Management of Personal Finance Business of Commercial Banks” has been adopted at the 33rd Chairman meeting of China Banking Regulatory Commission. Implemented since 1 November 2005.

9 Issued by the State Office of the State Council of China [2013] No. 107: Notice on Relevant Issues Concerning Strengthening Shadow Banking Regulation. The second paragraph of Article 3: “Focus on Improving the Supervision System and Measures".

10 “The Guiding Opinions of the General Office of the CBRC on the Risk Supervision of Trust Companies” (Issued by CBRC [2014] No. 99) stipulated that the capital pool is the business of investing in non-standard assets. It leads to the business where the source of funds and the use of funds cannot correspond one by one, as well as maturity mismatch between the source of funds and the use of funds. (Long-term use of short-term funds, maturity mismatch).

11 “Guiding Opinions of CBRC on Risk Prevention and Control of Banking Industry” (Issued by CBRC [2017] No. 6), “Circular of CIRC on Strengthening the Business Supervision of Portfolio Insurance Asset Management Products” (Insurance and supervision funds [2016] No. 104) believe that the capital pool business adopts the operation mode of “rolling sale, collective operation, maturity mismatch and separate pricing”, which has not been separately established or is not independently accounted for.

12 On 28 September 2018, The China Banking and Insurance Regulatory Commission issued “The Measures for the Supervision and Administration of Financial Business of Commercial Banks” (2018 No. 6).

13 On 2 December 2018, The China Banking and Insurance Regulatory Commission issued “The Management Measures for Financial Management Subsidiaries of Commercial Banks” (2018 No. 7).

14 Section 45 of “The Securities Investment Fund Law”. On September 2016, “The Securities Investment Fund Law” was adopted at the fifth meeting of the Standing Committee of the 10th China National People's Congress.

15 The case of Anxin Trust promises a rigid payment of 400 million financial products. On December 31, 2020, The Hunan Provincial Higher People's Court ruled that the rigid payment commitment is invalid. This is the first case in China's court that ruled the “rigid payment” commitment is invalid.
